# A Narrative Review on the Prevalence of *Plasmodium falciparum* Resistance Mutations to Antimalarial Drugs in Rwanda

**DOI:** 10.3390/tropicalmed10040089

**Published:** 2025-03-29

**Authors:** Muharib Alruwaili, Abozer Elderdery, Emad Manni, Jeremy Mills

**Affiliations:** 1 Department of Clinical Laboratory Sciences, College of Applied Medical Sciences, Jouf University, Sakaka 72388, Saudi Arabia; emena@ju.edu.sa; 2 School of Medicine, Pharmacy and Biomedical Sciences, University of Portsmouth, Portsmouth PO1 2DT, UK; jeremy.mills@port.ac.uk

**Keywords:** malaria, *Plasmodium falciparum*, *pfcrt*, *pfmdr1*, *pfdhfr/pfdhps*, artemisinin, Rwanda

## Abstract

Malaria has been and remains a significant challenge in Africa and other endemic settings. Roughly, 95% of global morbidity and mortality due to malaria occurs within African populations and affects millions of individuals, especially those living in sub-Saharan countries, predominantly due to disease complications. Cultural factors such as unawareness of and disinterest in using recommended preventive tools and combating the primary host (i.e., the female Anopheles mosquito) play a significant role. This host transmits the malaria-causing *Plasmodium* parasite by biting an infected individual and spreading it to humans. The current overview focuses on the molecular markers associated with antimalarial drug resistance in *Plasmodium falciparum* (*P. falciparum*) in Rwanda, considered an exemplar of sub-Saharan countries where malaria is prevalent and effective policies on the development of malaria treatment, approved recently by WHO in 2025, have been adopted. The prevalence of mutations in key resistance genes, including *pfcrt*, *pfmdr1*, and *pfdhfr/pfdhps*, are linked to resistance against common antimalarial drugs such as chloroquine and sulfadoxine-pyrimethamine (SP). In addition, the *Plasmodium falciparum* kelch13 (*pfk13*) gene is linked to resistance against artemisinin, as its mutations can cause delayed parasite clearance and treatment failure. Despite changes in therapeutic use policies owing to high prevalence of variant alleles, which reduce the drug’s efficacy resistance to SP, the gene persists in Rwanda. Malaria parasites are becoming more resistant to chloroquine, leading to diminished effectiveness and slower recovery or treatment failure. Surveillance data reported from several studies provide crucial insights into the evolving trends of resistance markers and are vital for guiding treatment protocols and informing therapeutic use policy decisions. It is important that we continue to maintain and develop the effectiveness of malaria prevention strategies and treatments, due to the multiple types of resistance found in the population.

## 1. Introduction

In 2023, around 263 million cases of malaria and 597,000 malaria-related fatalities were documented globally [1]. With Southeast Asia and parts of the Western Pacific, Africa shares the highest malaria incidence and remains the region with the highest disease burden, accounting for more than 95% of cases and deaths globally. Although five Plasmodium species can cause clinical malaria in humans (specifically: *P. falciparum*, *P. vivax*, *P. ovale*, *P. malariae*, and *P. knowlesi*), *P. falciparum* infections pose the greatest threat due to factors including infection severity and mortality, treatment resistance, high transmission and parasite lifecycle, and nearly all human malaria-related deaths are attributed to this species [2]. Drug resistance poses a major obstacle for malaria control. *P. falciparum* has exhibited the capacity to acquire resistance to numerous antimalarial drugs, including chloroquine (CQ), sulfadoxine, and pyrimethamine (SP). Mechanisms underlying resistance to these drugs have been identified and are associated with the presence of certain genetic polymorphisms [3,4,5]. Resistance to antimalarial drugs is linked to genetic polymorphisms in Plasmodium falciparum, reducing drug efficacy. Key polymorphisms include: *pfcrt* mutations (K76T) for chloroquine resistance [6], kelch13 mutations for artemisinin resistance, [7] dhfr mutations for sulfadoxine-pyrimethamine resistance, [4,5] pfatp6 mutations for quinine resistance, and *pfmdr1* mutations for resistance to both mefloquine and lumefantrine, especially in combination with artemether [8]. These mutations contribute to reduced drug effectiveness and treatment challenges. Mutations in *pfmdr1* and *pfcrt* affect *P. falciparum* resistance to antimalarial drugs [9], with some studies reporting that *pfmdr1* mutations confer resistance to chloroquine/amodiaquine, but may enhance susceptibility to lumefantrine/mefloquine, exhibiting an inverse relationship [9,10,11], which can make determining the correct therapeutic choice challenging without knowing the particular mutational spectrum. Similarly, chloroquine efficacy is decreased by *pfcrt* mutations. These mutations also contribute to resistance mechanisms against different drugs [11,12].

Due to widespread resistance against CQ and SP, ACT (artemisinin-based combination therapies) were endorsed as a first-line treatment [13]; however, SP was used as an intermittent preventive therapy for pregnant women (IPTp-SP) until 2008, at which time the drugs were withdrawn in Rwanda completely [14,15]. The waning efficacy of ACT in Southeast Asian countries has been noted since 2008, and it is characterized by a delay in parasite clearance (also referred to as slow parasitemia) [16,17,18]. The mechanism underlying resistance to ACT is attributed to certain single nucleotide polymorphisms (SNPs) in the propeller domain of the *P. falciparum* Kelch-13 (*pfk13*) gene and was initially identified in southeast Asia [19]. Genetic analysis of *P. falciparum* isolates has found that mutations at *pfk13* Y493H, *pfk13* R539T, *pfk13* I543T and *pfk13* C580Y are associated with artemisinin resistance by delaying clearance and enabling longer parasite survival [20]. Mutations conferring resistance and/or delay in parasite clearance have been observed in East Africa, including Rwanda, since 2010 [21,22,23]. Certain *pfk13* genotypes, such as *pfk13* R561H, *pfk13* C469Y, and *pfk13* A675V, were detected in Rwanda and found to be associated with slow parasitemia in vivo as well as reduced vulnerability in vitro [24,25,26]. A change in drug policy may influence the vulnerability of *P. falciparum* populations to certain drugs as this may reduce the selective pressure on parasites [14,27,28].

Since it is not ethically feasible to conduct clinical trials to estimate drug efficacy in areas with established high treatment failure rates, detection of resistance markers may serve as the main evidence to justify the reintroduction of previously withdrawn medications. Data on resistance markers can help identify whether resistance to a drug is present in a population and how it evolves over time, allowing for a better understanding of treatment failures to provide valuable information for rational drug policy formulation, managing malaria cases and the development of effective methods to alleviate the impact of drug resistance [29]. Additionally, in vitro resistance testing can be conducted under controlled conditions on isolated pathogens taken from patients whose treatment has failed to find whether this resulted from the development of resistance. These alternatives could collectively support decisions around reintroducing medications without the need for large-scale clinical trials, focusing instead on molecular evidence, patient data, and real-world effectiveness [30].

A 2003 study in Malawi was unable to detect *P. falciparum* chloroquine resistance transporter (*pfcrt* K76T) mutation in isolates, in contrast to the 85% prevalence reported in 1992 [27]. This suggests that sensitive parasite populations may re-emerge after drug pressure is removed. A slower rate of recovery to CQ sensitivity has been seen in Rwanda, implying that other factors may contribute to the rate of sensitive parasite re-emergence [14].

Therefore, in this review, we summarize the published data relating to molecular markers associated with antimalaria resistance in Rwanda. This can offer a refined understanding of antimalaria drug resistance monitoring in Rwanda that will help decision-making for policy development and management.

## 2. Study Selection Method

This review study was conducted on the drug resistance patterns to *P. falciparum* in Rwandan. It includes randomized controlled trials and both prospective observational and retrospective studies and concentrates on resistance to *P. falciparum*, as it is the most common drug-resistant strain in Rwanda and across sub-Saharan Africa. Antimalarial drug treatments, both first and second-line, for example, artemisinin-based combination therapies (ACTs), chloroquine, sulfadoxine-pyrimethamine, and quinine, were the focus along with *pfcrt*, *pfmdr1*, *pfdhfr*, *pfdhps* and *pfk13* genetic markers. All published studies reporting on the prevalence, presence, mechanisms, or resistance patterns were considered. This encompassed both molecular studies (gene mutations, genetic markers) and clinical studies (treatment failure, reduced efficacy). The most current data were captured from studies published in the last three decades by the World Health Organization (WHO), Malaria Publications, PubMed, Web of Science, African Journals Online, Rwanda Ministry of Health Reports and Guidelines and National Institutes of Health (NIH) Malaria Databases on anti-malarial resistance in Rwanda. The sources were systematically searched using a combination of terms, including (malaria; *P. falciparum*; molecular marker, *pfcrt*, *pfmdr1*, *pfdhfr*, *pfdhps* and *pfk13*) or (Rwanda). Articles related to *P. falciparum* were screened, and only those containing the resistance markers of interest proceeded to analysis. Data were categorized by geographic regions within Rwanda, antimalarial drug classes, and the temporal trends in resistance. Analysis identified implications for treatment strategies.

In vitro studies were excluded, as were animal model studies (those involving non-human subjects). Also rejected were those focusing on *P. vivax* or other malarial species, those on non-antimalarial treatment drugs (e.g., antibiotics), and those for unrelated infectious diseases. Moreover, also excluded were those lacking data on drug resistance and those concerned with drug efficacy but not assessing resistance mechanisms. Those with unclear study design, unreliable data reporting, or high risk of bias, as well as those not showing either relevant or full data, were also excluded.

For classifying patterns, trends, and overall resistance status, the studies were categorized by drugs used set against trends in resistance mechanism, the former being specific drugs (e.g., ACTs, chloroquine, quinine, sulfadoxine-pyrimethamine) or combinations, the latter including location and time. This allowed for a deeper understanding of the molecular and/or genetic basis of *P. falciparum*’s resistance and helped to identify regional differences in drug resistance patterns, providing more localized insights for treatment strategies.

## 3. Malaria in Rwanda

Rwanda is a small, land-locked country located in east central Africa covering a land area of about 24,670 square kilometers and a home of 12,955,763 individuals. The entire population of Rwanda is at risk of malaria, although malaria transmission levels show spatial heterogeneity, which has been associated with the variation in malaria prevention measures placed in different settings in the country. Rwanda is categorized into four malarial zones, which are based on several factors, including altitude, level of transmission, and vector density. *P. falciparum* is the predominant species in Rwanda and accounts for around 97% of the total malaria cases, although *P. ovale* and *P. malariae* have been detected as well. The primary vector that transmits malaria is *Anopheles gambiae* [15].

The first malaria case in Rwanda was documented in 1917 and since then malaria has become prevalent [31]. Rwanda achieved a significant accomplishment in terms of reducing malaria cases between 2005 and 2011 where the prevalence of malaria cases and deaths declined by 86% and 74%, respectively. However, an upsurge in malaria cases was reported in the following years, with an increase of 564,407 cases in 2012 to 4,746,985 cases in 2017—which shows an increase of more than eight-fold. Over 79% of the malaria cases were reported in the Southern and Eastern provinces. In the Southern province specifically, there was a 13-fold increase in malaria cases (132,108 in 2012 to 1.8 million in 2016). The rise in malaria cases has been linked to multiple factors, including inadequate distribution of insecticide-treated nets (ITNs) and indoor residual spraying (IRS) efforts (both the availability of nets and the quality/application of the insecticide used), emerging insecticide resistance, and increased irrigated agriculture. Malaria cases in Rwanda between 2019 and 2020 dropped by more than 50% (198 cases per 1000) compared to 2017 [15].

## 4. Current Antimalarial Drug Therapies

Due to increasing resistance to the previously effective treatment CQ, it was replaced by SP in 2001 [32]. Between 2001 and 2006, SP-AQ (sulfadoxine-pyrimethaminem and amodiaquine) was adopted as a first choice of treatment for uncomplicated *P. falciparum* infection [14]. Since 2006, Rwanda has shifted from SP-AQ to ACT as the prevailing treatment protocol for uncomplicated malaria [13]. Artemether-lumefantrine (AL) serves as the first-line treatment while dihydro-artemisinin-piperaquine is the second-line treatment. AL is the first-line treatment for pregnant women throughout all three trimesters, whilst severe malaria in all patients except pregnant women is treated with parenteral artesunate.

## 5. Prevalence of Resistance Marker in *P. falciparum* in Rwanda

The first study that reported resistance markers in *P. falciparum* in Rwanda was carried out by Karema and coworkers in 2005–2006 among children aged between 6 and 59 months. The study only reported the molecular markers linked with SP resistance [33]. The samples were collected as part of a randomized clinical trial to assess the safety and effectiveness of artesunate combined with chlorproguanil-dapsone and SP combined with AQ for the treatment of uncomplicated *P. falciparum* malaria in children less than five-years old [34]. Both of the genes linked to SP resistance (*pfdhfr* and *pfdhps*) were successfully genotyped in 725 samples. N51I, C59R, S108N, and I164L were the four SNPs in the *pfdhfr* gene and S436A, A437G, K540E, and A581G were among the four SNPs in the pfdhps gene that were genotyped. Table 1 shows the prevalence of these SNPs in addition to triple *pfdhfr* (N51I, C59R, S108N), double *pfdhps* A437G, K540E or K540E and A581G, quintuple (*pfdhfr* N51I, C59R, S108N and *pfdhps* A437G, K540E) and sextuple (*pfdhfr* N51I, C59R, S108N and *pfdhps* A437G, K540E or K540E and A581G) SNPs.

In 2010, a study in Rwanda’s Southern province investigated the frequency of molecular marker SNPs in *P. falciparum* in children under the age of five [32]. There were 104 *P. falciparum* isolates, which were genotyped for 16 SNPs. These include A16V, C59R, N51I, I164L and S108N in the *pfdhfr* gene; S436A, K540E, A437G, A613S and A581G in the *pfdhps* gene; N86Y, N1042C, Y184F, D1246Y, S1034C in the *pfmdr1* gene and K76T in the *pfcrt* gene (see Table 1).

The incidence and distribution of *P. falciparum* molecular markers of resistance to SP and CQ following 14 and 7 years of withdrawal of these drugs, respectively, was reported in 2015. The investigation was conducted in the Western and Eastern provinces representing high and low transmission zones [14]. There were 399 *P. falciparum* isolates included, and a total of 14 SNPs in four different genes were genotyped, including N51I, S108N, C59R, and I164 in the *pfdhfr* gene; A437G, A581G, K540E, and A613S in the *pfdhps* gene; N86Y, Y184F, N1042C, S1034C, and D1246Y in the *pfmdr1* gene; K76T in the *pfcrt* gene.

More recently, a 2016–2018 study reported on nine SNPs linked with resistance to sulfadoxine and pyrimethamine [29], including N51I, S108N, C59R, and I164 in the *pfdhfr* gene and S436A, A581G, K540E, A437G, and A613S in the *pfdhps* gene. The study included only pregnant women who attended antenatal care clinics in the Southern province of Rwanda [35]. There were 148 placental samples genotyped successfully for both *pfdhfr* and *pfdhps* genes.

**Figure 1:**
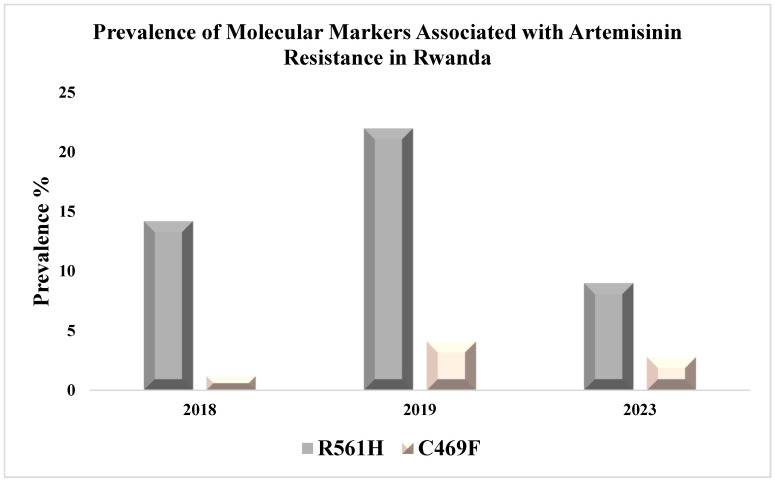
Molecular Markers (R561H and C469F) Associated with Artemisinin Resistance in Rwanda.

The most recently reported data on artemisinin resistance were published in 2023 from samples collected from the same year and showed that the prevalence of *pfk13* R561H, A675V, and C469F were (9% 19/212; 5.7% 12/212; 2.8% 6/212), respectively, which almost double the frequency from the same area compared to a 2019 study, as summarized in Table 1 and Figure 1 [21,36]. Three *pfmdr1* N86, 184F and D1246 genotypes have been reported, and the frequencies of these alleles were 98% 97/99; 44.4% 44/99; ~86% 85/99, respectively [36]. The *pfmdr1* N86 increased from 60.6% in 2010 to 98% in 2023 [32,36]. Lumefantrine tolerance and artemether-lumefantrine treatment failure has been associated with *pfmdr1* N86 [37]. Between 2018 and 2019, a study conducted at two study sites in Kigali City, reported *pfk13* R561H, *pfk13* P674L, and *pfk13* C469F in 73 patients at frequencies of ~22%, 1%, and 4%, respectively. The study also reported two novel mutations as well: *pfk13* Q661E and *pfk13* P667S [23]. The reported data of all validated resistance markers associated with artemisinin resistance in Rwanda are shown in Table 2.

## 6. Discussion

Resistance markers can provide valuable information regarding spatiotemporal trends in drug resistance. In addition, data on resistance markers can be used to guide treatment policies and monitor parasite susceptibility following drug policy changes. However, determining the predictive usefulness of molecular markers for clinical treatment outcomes has been more difficult due to the fact that there are other factors besides the intrinsic parasite resistance that impact outcomes [29].

Rwanda transitioned from SP+AQ to ACT as the first-line therapy for uncomplicated malaria in 2006 and discontinued intermittent preventive treatment for pregnant women with sulfadoxine-pyrimenthamine (IPTp-SP) in 2008 [14,15]. SNPs in the *pfdhps* and *pfdhfr* genes, respectively, are linked to resistance to SP. Five mutations in the *pfdhfr* gene were linked to pyrimethamine resistance in *P. falciparum,* and these mutations include A16V, N51I, S108N, C59R, and I164L; the latter SNP is linked to significant resistance to SP [4]. Mutations at A437G, S436A, A581G, K540E, and A613S in the *pfdhps* gene are linked to sulfadoxine resistance [5]. Haplotype combinations comprised of the triple mutations in *pfdhfr* (N51I, C59R and S108N/T) and double mutations in *pfdhps* (A437G and K540E), commonly known as the quintuple mutant genotype, are predominant in East Africa and are linked to clinical and parasitological SP treatment [39]. Despite the discontinuation of SP in Rwanda as first-line therapy in 2006 and as IPTp in 2008, a significant prevalence of quintuple (75%) mutants persisted after 8 to 10 years of drug withdrawal [35]. Data from Rwanda showed that the quintuple genotype increased over time from 31.3% in a 2005–2006 study to 75% in a 2016–2018 study [33,35]. A similar pattern was found in a report from the Democratic Republic of Congo, which borders Rwanda, indicating an increase in *pfdhps* K540E mutation, a proxy marker for the quintuple haplotype, from 2% in 2007 to 13% in 2013 [40]. The enduring prevalence of high-level SP resistance in Rwanda, persisting years after SP withdrawals has been observed elsewhere, including Uganda and Tanzania; although, in Ethiopia, a reduction in *pfdhfr* and *pfdhps* mutant alleles has been reported [41,42,43]. The sustained high-level resistance to SP observed in Rwanda, despite the fact that there was a presumed absence of SP pressure in the parasite population for more than 8 years, may be due to the ongoing use of SP or other related drugs such as trimethoprim and sulfamethoxazole, which have been suggested to exhibit a cross resistance with SP, or the gene flow from neighboring countries [44]. The rise in the predominance of the quintuple genotype has been concomitant with enhanced utilization of IPTp-SP.

CQ and AQ resistance has been associated with mutations in the *pfcrt* and *pfmdr1* genes [3,45]. In Rwanda, only two studies have reported on the *pfcrt* and *pfmdr1* genes [14,32]. From the available data, variant *pfcrt* K76T was reported in 2010, and it showed that 74% (N = 104) of the isolate carried mutant alleles. In a 2015 study, the figure was 49% (N = 399), though in a different region, an approximate 25% recovery rate was suggested even after a decade following CQ withdrawal [14,32]. In contrast, high recovery rates of CQ-susceptible alleles have been observed in Malawi, where the wildtype allele *pfcrt* K76T reverted after 12 years of CQ withdrawal. The variant *pfcrt* K76T in Malawi diminished over time following CQ replacement with SP where the mutation disappeared from isolates collected in 2001 compared to 85% in 1992 [27]. The re-emergence of wildtype alleles in the absence of drug pressure has a clinical implication as these parasites have now become sensitive to CQ, as has been shown by improved CQ efficacy in clinical trials and in vitro studies [28]. Other countries have reported high rates of recovery of CQ-susceptibility [46,47]. The slower recovery rates observed in Rwanda could be attributed to the ongoing use of CQ or other analogous drugs such as AQ, which have been associated with limited recovery of CQ-susceptible alleles [48], the level of CQ resistance at the baseline, transmission intensities, and the actual time since drug policy change.

Mutations in the *pfmdr1* gene are linked to resistance to quinine, CQ, mefloquine, and artemisinin [10,11]. The variant alleles for *pfmdr1* were reported only in two studies (2010 and 2015) [14,32]. In 2010, mutations in the pfmdr1 gene were reported, including N86Y, Y184F, S1034C, N1042C and D1246Y. However, a 2015 study reported just three mutations, which included N86Y, Y184F, and D1246Y, whilst no mutant alleles were detected at S1043C and N1042C. The predominant allele was *pfmdr1* Y184F (52%) followed by N86Y (38%) in 2010 [26]. Other mutant alleles, *pfmdr1* D1246Y, S1034C, and N1042C, have been reported at prevalences of 12%, 2.9%, and 2.9%, respectively. The mutations at *pfmdr1* Y184F and N86Y were detected in 2015, but an increase was seen in *pfmdr1* Y184F 59% in 2015 compared to 52% in 2010. In contrast, a decrease was observed in *pfmdr1* N86Y where 19.1% of isolates carried the mutation in 2015 compared to 38% in 2010, suggesting opposite selection may have occurred in these loci [14,32]. A relative increase was also reported in *pfmdr1* D1246Y from 12% in 2010 to 19.2% in 2015. The two mutant alleles S1034C, N1042C were not detected in 2015 in all genotyped samples [14]. It is believed that the mixed selection reported in *pfmdr1* could be attributed to the scale-up of AL, which has been demonstrated to select for CQ-susceptible alleles [49].

In Rwanda, molecular markers associated with a delay in parasite clearance and/or resistance to artemisinin are emerging and increasing over time, sending an alarm that the efficacy of ACT could be impacted. Fortunately, more recent studies have confirmed that the cure rates of artemether-lumefantrine (AL) exceed 93%, implying that the drug is still efficacious [22,42]. Evidence from East Africa demonstrated that AL susceptibility is inversely correlated with chloroquine and amodiaquine, as directional selection on the *pfcrt* and *pfmdr1* resistance was observed [50]. Interestingly, the *pfmdr1* N86 confers reduced susceptibility to lumefantrine and increased AL treatment failure [37,51]. Although the actual role of *pfk13* and *pfmdr1* polymorphisms in ACT resistance is far from being understood, systematically monitoring these genomic determinants is crucial so that alternative regimens can be implemented. The rise and expansion of the *pfk13* R561H variant in Rwanda is notable and requires effective strategies to contain the spread of resistance across the country and into neighboring countries. As a consequence, Rwanda could see a rise in resistance to ACT partner drugs leading to higher treatment failure rates such as those seen in Southeast Asia.

## 7. Conclusions

Malaria remains a challenging public health problem in Rwanda despite the tremendous progress made in reducing malaria morbidity and mortality. Monitoring and tracking molecular markers are essential for malaria management and to help design strategies to mitigate risk and reduce incidences. The data presented here represents limited geographic regions from which samples were collected. Although in Rwanda the entire population is at risk of malaria, transmission exhibits spatial heterogeneity. Therefore, data on antimalarial drug resistance should be reported from all endemic regions of the country to better track emerging resistance and monitor the efficacy of malaria treatment.

## Figures and Tables

**Table 1:** The prevalence of SNPs affecting antimalarial therapeutic efficacy in provinces in Rwanda (2005–2018) [14,32,33,35]. “*” means Not Detected.

		Eastern and Western Provinces					Southern Provinces	
		2005/6 (*N* = 725) [33]		2010 (*N* = 104) [32]	2015 (*N* = 399) [14]		2016/18 (*N* = 148) [35]	
Gene	SNP	Rukara, (%)	Mashesha, (%)	Huye, *n* (%)	Mubuga, *n* (%)	Ruhuha, *n* (%)	Huye, *n* (%)	Kamonyi, *n* (%)
*pfdhfr*	I164L	11.4	ND^2^	ND	ND	19 (10.2)	ND	ND
	A16V			ND *				
	N51I			103 (99)	185 (99.5)	190 (100)	44 (96)	97 (98)
	C59R			78 (75)	184 (96.3)	160 (84.2)	42 (89)	95 (94)
	S108N			103 (99)	189 (100)	190 (100)	46 (98)	101 (100)
*pfdhps*	S436A			(1)			32 (74)	64 (69)
	A437G	97	80	98 (96)	169 (93.9)	169 (91.9)	39 (91)	83 (89)
	K450E	56.5	47	97 (94)	170 (94.4)	173 (94.5)	39 (91)	84 (89)
	A581G	60	29	64 (63)	39 (21.7)	49 (26.8)	12(31)	36 (41)
	A613S	ND *	ND	ND	ND	ND	2 (5)	2 (2)
Triple *pfdhfr*	N51I-C59R-S108N	83.9	49.3	83.90%	179 (96.2)	160 (84.2)	40 (85)	91 (90)
Double *pfdhps*	A437G-K540E			36.20%	166 (92.2)	168 (91.8)	39 (83)	83 (82)
Quintuple	*pfdhfr* N51I, C59R, S108N-*pfdhps* A437G, K540E	31.3	23.1	20 (19.2)	153 (74.6)	143 (73.7)	35 (74)	76 (75)
Sextuple	*pfdhfr* N51I, C59R, S108N-*pfdhps* A437G, K540E, A581G	46.5	14.2	52 (50)	31 (15.1)	42 (21.7)	11 (23)	30 (30)
*pfcrt*	K76T			77 (74)	86 (43.7)	105 (55)		
*pfmdr1*	N86Y			40 (38)	33 (43.7)	40 (21.5)		
	Y184F			56 (52)	100 (57.1)	107 (62.6)		
	S1034C			3 (2.9)	ND	ND		
	N1042C			3 (2.9)	ND	ND		
	D1246Y			13 (12)	35 (17.8)	37 (20.8)		

**Table 2:** The prevalence of molecular markers in *P. falciparum* K13 marker associated with a delay in parasite clearance and/or resistance to artemisinin.

Year	Resistance Marker	Prevalence n, (Sample Size)	References
2010	None	ND	[38]
2014	V555A, A626S	2 (81)	[38]
2015	P574L, D648H, A675V	3 (66)	[38]
2018	R561H, P574L, C469F	36, 2, 3 (254)	[22]
2019	R561H, P574L, C469F	16, 1, 3 (73)	[23]
2023	R561H, A675V, C469F	19, 12, 6 (212)	[36]

## Data Availability

No new data were created or analyzed in this study.

## Abbreviations

The following abbreviations are used in this manuscript:

SP	Sulfadoxine-pyrimethamine
ACTs	Artemisinin-based combination therapies
CQ	Chloroquine
AQ	Amodiaquine
SNPs	Single nucleotide polymorphisms
*pfk13*	*P. falciparum* Kelch-13
*pfmdr1*	*P. falciparum* multidrug resistance
IPTp-SP	Intermittent preventive therapy for pregnant women
*pfcrt* K76T	*P. falciparum* chloroquine resistance transporter
ITNs	Insecticide-treated nets
AL	Artemether-lumefantrine
